# Time‐Dependent Effects of Low‐Intensity Pulsed Ultrasound on Apoptosis and Autophagy in Malignant Melanoma Stem Cells

**DOI:** 10.1111/jcmm.70687

**Published:** 2025-06-25

**Authors:** Omer Dikici, Berrin Ozdil, Taha Kadir Yesin, Aylin Dikici, Yasemin Adalı, Huseyin Aktug

**Affiliations:** ^1^ Department of Stem Cell, Institute of Health Sciences Ege University Izmir Turkiye; ^2^ Private Metropol Zubeyde Hanım Medical Centre Clinic of Physical Medicine and Rehabilitation Izmir Turkiye; ^3^ Department of Histology and Embryology, Faculty of Medicine Suleyman Demirel University Isparta Turkiye; ^4^ Department of Physics, Faculty of Science Izmir Institute of Technology Izmir Turkiye; ^5^ Department of Histology and Embryology, Faculty of Medicine Ege University Izmir Turkiye; ^6^ Clinic of Physical Medicine and Rehabilitation Tepecik Training and Research Hospital Izmir Turkiye; ^7^ Centre for Public Health, School of Medicine, Dentistry and Biomedical Sciences Queen's University Belfast Belfast UK; ^8^ Department of Biophysics Pamukkale University Medical Faculty Denizli Turkiye

**Keywords:** autophagy, apoptosis, mechanobiology, cancer stem cell, LIPUS, melanoma

## Abstract

Cancer stem cells (CSCs) in malignant melanoma contribute to therapeutic resistance and tumour recurrence. While low‐intensity pulsed ultrasound (LIPUS) has been proposed as a non‐invasive strategy to induce cell death, its effects on CSC‐specific apoptotic and autophagic responses remain unclear. This study aimed to explore the time‐dependent effects of LIPUS on apoptosis and autophagy in CD133+ melanoma CSCs and CD133− non‐stem melanoma cells. Human melanoma cells (CHL‐1) were sorted via FACS into CD133+ and CD133− populations. Cells were exposed to LIPUS (1 MHz, 20% duty cycle, 1 W/cm^2^) for 1, 5, and 10 min. Protein expression levels of Caspase‐3, Caspase‐8, mTOR, and LC3 were evaluated via immunofluorescence and quantified by image‐based analysis. Both cell populations showed significant increases in Casp3, Casp8, mTOR, and LC3 intensities following LIPUS application. Notably, CD133+ cells exhibited delayed but sustained increases in Casp3 and LC3 expression, while CD133− cells responded more rapidly. mTOR activity demonstrated distinct temporal dynamics between the two groups, suggesting differential modulation of autophagy‐related pathways. LIPUS triggers temporally distinct apoptotic and autophagic responses in melanoma CSCs and non‐stem cancer cells. These findings suggest a potential therapeutic avenue to selectively disrupt CSC survival mechanisms using mechanical stimulation.

## 1 Introduction

Malignant melanoma (MM) is a cancer type originating from these melanocyte cells. Its most significant site of occurrence is the skin (cutaneous melanoma), but it can also manifest in other tissues containing melanocytes (mucous membranes, eyes, internal organs, and the central nervous system) [1]. The incidence of cutaneous melanoma worldwide has been steadily increasing over the years, and especially in western societies, the lifetime risk of melanoma has reached a rate of 1 in 50 [2]. The incidence of melanoma in Europe is < 25 cases per 100,000 people; in the United States (US) it is 30 cases per 100,000 people; and in Australia, which is the region with the highest prevalence of melanoma worldwide, it reaches a rate of 60 cases per 100,000 people [3, 4]. Studies on different chemotherapeutic drugs in melanoma are ongoing, both during the treatment process and in post‐treatment follow‐up [4].

In previous years, numerous studies have reported the presence of cells within cancer that exhibit stem cell‐like properties such as self‐renewal and differentiation abilities called cancer stem cells. Cancer stem cells (CSCs) were initially identified within the haematopoietic system. Bonnet et al. isolated CSCs in acute myeloid leukaemia and successfully transplanted them into immunodeficient mice. These cells were characterised by the cell surface markers CD34+/CD38− [5]. CSCs share similarities with normal stem cells in terms of stem cell properties. However, CSCs distinguish themselves from normal stem cells by their role in initiating and promoting tumour growth, as well as their resistance to radiation, chemical agents, and apoptosis [6]. The origins of CSCs are not yet fully understood. It has been proposed that they may arise through mutations in adult stem cells, but this is not always the case. It is also believed that CSCs can potentially originate from the oncogenic transformation of transit amplifying cells, progenitor cells, or differentiated cells [7].

Melanoma is a tumour that contains heterogeneous cell populations. The presence of heterogeneous cell groups can contribute to tumorigenicity and chemoresistance [8]. In 2005, Fang et al. discussed the presence of cells exhibiting stem cell‐like properties in human melanoma [9]. These cells were found to form spheroids and exhibited a high expression of the CD20+ surface marker. However, their classification as true stem cells was primarily based on their abilities to self‐renew, differentiate, and form tumours [7]. The CD133+ surface marker, which is commonly used to identify stem cells in some cancer types, has also been found to be abundant in stem cell‐like cells isolated from melanoma patients [10]. CD133 is a transmembrane protein with a glycoprotein structure. CD133+ cells have an increased potential for tumour formation. Melanoma contains a significantly higher percentage of CSCs compared to other tumour types (27% vs. 0.0001%) [11]. This situation suggests that it could be a promising choice for use in research.

Stem cells and their microenvironments interact with mechanical forces to influence cell behaviour and fate determination [12]. Different mechanical forces lead to the generation of distinct cellular responses. Furthermore, the same mechanical force can lead to different cellular outcomes depending on the cell type, geometry, type, chromosomal condensation, and differentiation stage [13]. Stem cells respond to mechanical forces with various cellular processes such as proliferation, migration, self‐renewal, and differentiation. Examples of mechanical forces include mechanical stretching (tension, stretching, elongation), mechanical loading, vibration, mechanical compression, pressure, and sound waves [14].

Ultrasonography is a form of mechanical energy. Mechanical vibrations at increasing frequencies are known as sound energy. The human ear can perceive sounds in the frequency range of 15–20,000 Hz. Mechanical vibrations above this upper limit are referred to as ultrasound. Ultrasound can be used for imaging and therapeutic purposes. The frequencies used in therapy typically fall within the range of 0.7–3.3 Megahertz (MHz) [15]. Furthermore, the amount of energy applied per unit area, known as intensity, is also crucial for the effect of ultrasound, and its unit is W/cm^2^. In therapy, ultrasound is used in two modes: continuous and pulsed (intermittent, in cycles of 1:1–1:4). When continuous ultrasound is applied, sound waves penetrate the tissue, causing an increase in molecular vibration within the tissue, resulting in a rise in temperature [16]. As a result, continuous ultrasound is used in therapy with its thermal and mechanical effects, while pulsed ultrasound does not generate heat in tissues and is used for therapy based on its mechanical effects.

From a mechanobiological perspective, low‐intensity mechanical stimuli can influence cell fate decisions, including survival and death pathways. Low‐Intensity Pulsed Ultrasound (LIPUS) is a non‐thermal and non‐destructive acustic treatment method [17]. The United States Food and Drug Administration (FDA) has approved the use of LIPUS for non‐union fractures [18]. In recent times, the use of LIPUS has gained importance in cancer treatment and in other pathologies [19]. LIPUS is a non‐invasive method of stimulation that has gained attention in recent years, demonstrating beneficial impacts on neuromodulation, fracture repair, reducing inflammation, and regulating metabolism [20]. Apart from these, there are studies on the differentiation of stem cells [21, 22] and intracellular drug uptake in cancer studies. Furthermore, there are limited studies on apoptosis, autophagy and LIPUS in cancer cells [23, 24]. Regarding CSCs, studies on glioblastoma was carried out on the use of LIPUS and the investigation of drug effect and senescence [24, 25]. Therefore, there are no studies related to apoptosis and autophagy in both cancer stem cells and melanoma, so far.

The incidence of melanoma is expected to rise in the coming years due to climate change‐related factors such as increased exposure to ultraviolet (UV) radiation, rising ambient temperatures, and ecosystem disruptions that affect human health. Specifically, UV radiation, which is intensified by ozone layer depletion, is a known risk factor for malignant melanoma, potentially increasing CSC burden and challenging treatment outcomes. Furthermore, the heightened prevalence of environmental stressors, including heat stress and pollutant exposure, could exacerbate oxidative stress and DNA damage, potentially influencing CSC dynamics and treatment resistance. These considerations highlight the importance of adaptable and robust therapeutic strategies, such as LIPUS, in mitigating CSC‐driven resistance mechanisms in an era of changing environmental conditions. The temporal dynamics in apoptosis and autophagy induction observed in this study suggest that LIPUS could be particularly effective in targeting melanoma CSCs. This integration of innovative therapies and environmental awareness highlights a path forward for improving cancer treatment efficacy in a rapidly evolving global landscape.

## 2 Methods

### 2.1 Cell Culture

The CHL‐1 (ATCC CRL‐9446TM) non‐pigmented human melanoma cell line was cultured in MEM with Earle's Salts with L‐Glutamine (MEM‐A, Capricorn) growth medium supplemented with 10% fetal bovine serum (Biowest, S1810) and 1% Penicillin/Streptomycin (PS‐B, Capricorn). The CHL‐1 cell line was used at passages 6 and 8, while the CD133+ and CD133− cell lines obtained through flow cytometry were used up to passage 4.

### 2.2 Fluorescence‐Activated Cell Sorting (FACS)

Fluorescence‐Activated Cell Sorting (FACS) was used to isolate malignant melanoma CSCs by the CD133 marker. The cells were passaged, and then the supernatant was discarded. The cells were suspended in 1X PBS, and in a dark environment, 10 μL of CD133 (Miltenyi Biotec Ltd.) antibody was added, followed by pipetting to ensure cell mixing. After vortexing the cells, they were incubated at +4°C for 10 min, with aluminium foil wrapped around to prevent light exposure. During this process, unbound antibodies were removed by adding 5 mL of 1X PBS to the cells, followed by centrifugation at 1000 rpm for 5 min. Subsequently, the cells were passed through a 5 mL filtered tube. In this manner, the cells were prepared for FACS and sorted using a FACS Aria device (BD Pharmingen) to separate CD133+ MM CSCs [26].

Malignant melanoma cells were isolated using the FACS system at Izmir Biogenome Center. Cells labelled with CD133 antibody were designated as CD133+ MM cancer stem cells, while those not labelled were referred to as CD133− MM cancer cells, thus forming two experimental groups. Cells were prepared for the collection of CD133+ cells using flow cytometry and were separated into positive and negative cells based on gates p4 and p5.

### 2.3 LIPUS Application

**FIGURE 1:**
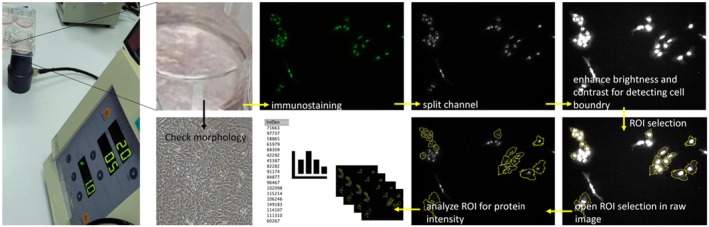
Following LIPUS application, cells were observed under a microscope and incubated for 48 h. The cells were then fixed and stained for the protein of interest. Images were analysed by first splitting the channels and adjusting the brightness and contrast to define cell boundaries and identify Regions of Interest (ROIs). The ROIs were analysed using raw 8‐bit images, and protein intensity levels were compared between groups after corrected total cell fluorescence method.

For LIPUS application, cells were diluted to 10^5^ cell/mL and were seeded into 6‐well polystyrene plates (Figure 1). In the study, two groups were established as MM CSCs (CD133+) and MM cancer non‐stem cells (CD133−). After incubation for 24 h at 37°C with 5% CO_2_, the cells were subjected to LIPUS treatment using the SONOPULS 190 device (Enraf Nonius, Amsterdam). For LIPUS treatment, the device's probe was placed under the cell culture plate, and ultrasound gel was applied to the top of the probe to facilitate the transmission of sound waves. The device was then activated [24], allowing controlled biophysical stimulation of the cells through acoustic energy. Both CD133+ and CD133− cell plates were subjected to 1 MHz, 20% duty cycle, and 1 W/cm^2^ LIPUS for 1, 5, and 10 min [27]. Before each application, a drop of water was placed on the tip of the probe to observe the vibrations generated, ensuring that the device was functioning. Additionally, during the application, vibrations occurring in the liquid inside the plates were periodically checked to confirm that the device was operational. The ultrasound‐treated cells were then incubated at 37°C with 5% CO_2_ for 48‐h.

### 2.4 Immunofluorescence

The cells were treated with 4% paraformaldehyde (Sigma P‐6148) at room temperature for 30 min for fixation. After this incubation, the cells were washed with 1X PBS (Thermo, 003002) to remove PFA. For samples that would undergo cell staining on the same day, the procedures continued. For samples planned for processing on subsequent days, the cells were stored at +4°C. To permeabilize the cells, they were incubated in 0.25% Triton‐X100 (Biotech, C34H62O11) prepared in advance for 5 min. To remove the Triton‐X100 solution, the cells were washed with 1X PBS. For blocking, the cells were incubated in 1% BSA (Chem Cruz, sc‐2323) at room temperature for 1 h. mTOR (Santa Cruz, sc‐517464), LC3 (Bioss, bs‐8878R), Casp3 (Bioss, bs‐2593R), and Casp8 (Bioss, bs‐0052R) antibodies were prepared by diluting them 1/100 in 1% BSA. The cells were incubated in the primary antibody solution overnight in a humid environment. To remove the excess antibody, the cells were washed three times with 1X PBS. The secondary antibody was prepared and allowed to interact with the cells for 1 h. After another three washes with 1X PBS, the cells were covered with a Fluoroshield Mounting Medium with DAPI (abcam, ab104139). Imaging was performed using an Olympus BX50 Fluorescence Microscope, and photographs were taken at 20X and 40X magnifications. All photographs were analysed using the Fiji/ImageJ program. Both cell populations showed significant increases in Casp3, Casp8, mTOR, and LC3 intensities following LIPUS application, measured relative to the 0‐min (baseline) group.

### 2.5 Statistics

In our study, we used Fiji/Image J, an image analysis software developed by the National Institutes of Health in Bethesda, MD, for conducting statistical analyses on immunofluorescence‐stained cells [28, 29]. This software was utilised to transform the uploaded images into black and white format by the split channel tool, with the green and blue channels specifically preserved for the current investigation. Green files were indicative of protein staining, while the blue files represented DAPI staining, highlighting the nuclear regions.

To facilitate data analysis, we employed the Region of Interest (ROI) tool within Fiji/Image J to delineate black and white images encompassing the cells, subsequently recording and storing the obtained data in an Excel spreadsheet. IBM SPSS Statistics 25.0 was the chosen tool for data analysis of protein intensities to compare groups. One‐way ANOVA followed by Tukey's post hoc test was used to determine statistical significance between groups. A minimum of 100 cells from at least ten photographs obtained from at least three different experiments were analysed. The raw data, which were RGB images, were converted to 8‐bit black and white images using the split channels function, focusing on the channel containing the protein image. The cell boundaries were manually drawn using the ‘freehand selection’ tool, and the intensities from this area were statistically compared between groups (Figure 1). The corrected total cell fluorescence (CTCF) method was applied, normalising the protein signal [30]. The normality of the data was assessed using the Shapiro–Wilk test, and Levene's test was used to evaluate the homogeneity of variance. Results were expressed as mean values ± standard deviation, with statistical significance denoted by *p < 0.05, **p < 0.01, and ***p < 0.001. GraphPad Prism 8.4.3 was the software utilised to generate graphical representations of the data, and experiments were conducted in triplicate for robustness.

## 3 Results

### 3.1 The Activation of Casp3 and Casp8 Shows Distinct Temporal Dynamics in Response to LIPUS in CD133+ and CD133− Melanoma Cells

**FIGURE 2:**
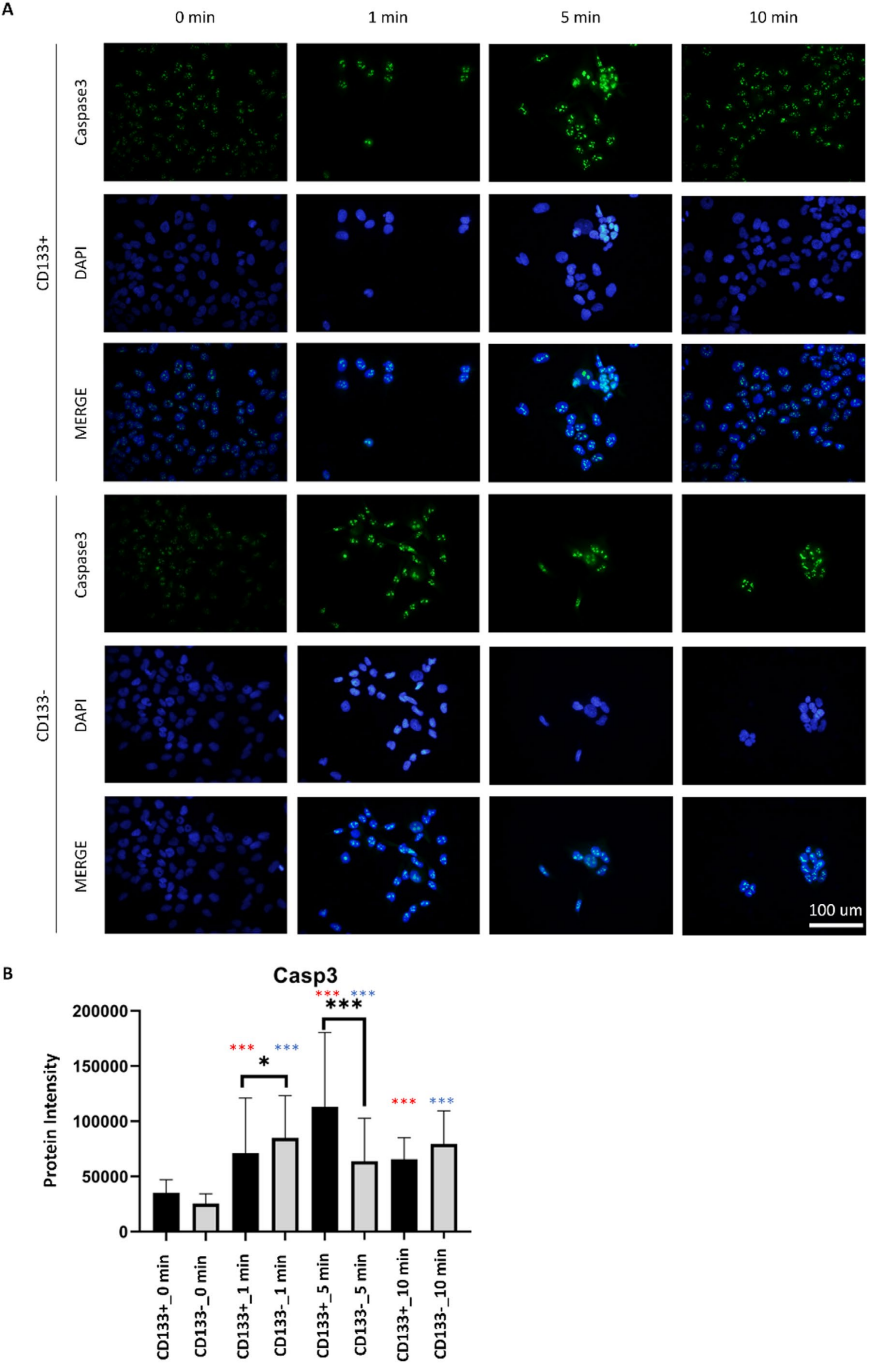
Casp3 protein immunostaining images (A) and quantitative comparison between groups (B). Statistical comparisons were performed between CD133+ and CD133− groups at each LIPUS application time point (black asterisks), as well as within each group relative to the untreated 0‐min baseline (red asterisks for CD133+, blue asterisks for CD133−). Data are shown as mean ± SD. Statistical significance was evaluated using one‐way ANOVA followed by Tukey's post hoc test. *p < 0.05, **p < 0.01, ***p < 0.001. No statistically significant difference was observed between the groups without LIPUS application in both CD133+ and CD133− cell groups. However, a significant difference was observed between the cell groups following 1‐min and 5‐min LIPUS applications (*p* < 0.05 and *p* < 0.001). In the CD133+ cell groups, Casp3 levels increased compared to the control group (0 min) (*p* < 0.001), with the maximum increase observed at 5 min of application. A similar pattern was observed in the CD133− cell group, with an increase in Casp3 protein immunostaining within the cells following LIPUS application (*p* < 0.001), with the maximum increase recorded at 1 min of application.

In the CD133+ group, LIPUS application resulted in a significant increase in Casp3 fluorescence at 1, 5, and 10 min compared to baseline (0 min) (*p* < 0.001), with the highest increase observed at 5 min (Figure 1). A similar pattern was observed in the CD133− group, where the fluorescence significantly increased (*p* < 0.001), peaking at 1 min. At baseline (0 min), there was no significant difference between the CD133+ and CD133− groups (*p* = 0.052). However, after LIPUS application, significant differences emerged at 1 and 5 min, with the CD133+ group showing delayed but sustained increases compared to the CD133− group. Casp8 fluorescence intensity significantly increased at 1, 5, and 10 min after LIPUS application (*p* < 0.001), with the highest intensity at 10 min in the CD133+ group (Figure 2). A similar trend was observed in the CD133− group, where LIPUS also induced significant increases (*p* < 0.001), peaking at 5 min. No significant differences were detected between the CD133+ and CD133− groups either before or after LIPUS application (*p* > 0.05).

### 3.2 LIPUS Induces Differential Activation of mTOR and LC3 Pathways in CD133+ and CD133− Melanoma Cells

**FIGURE 3:**
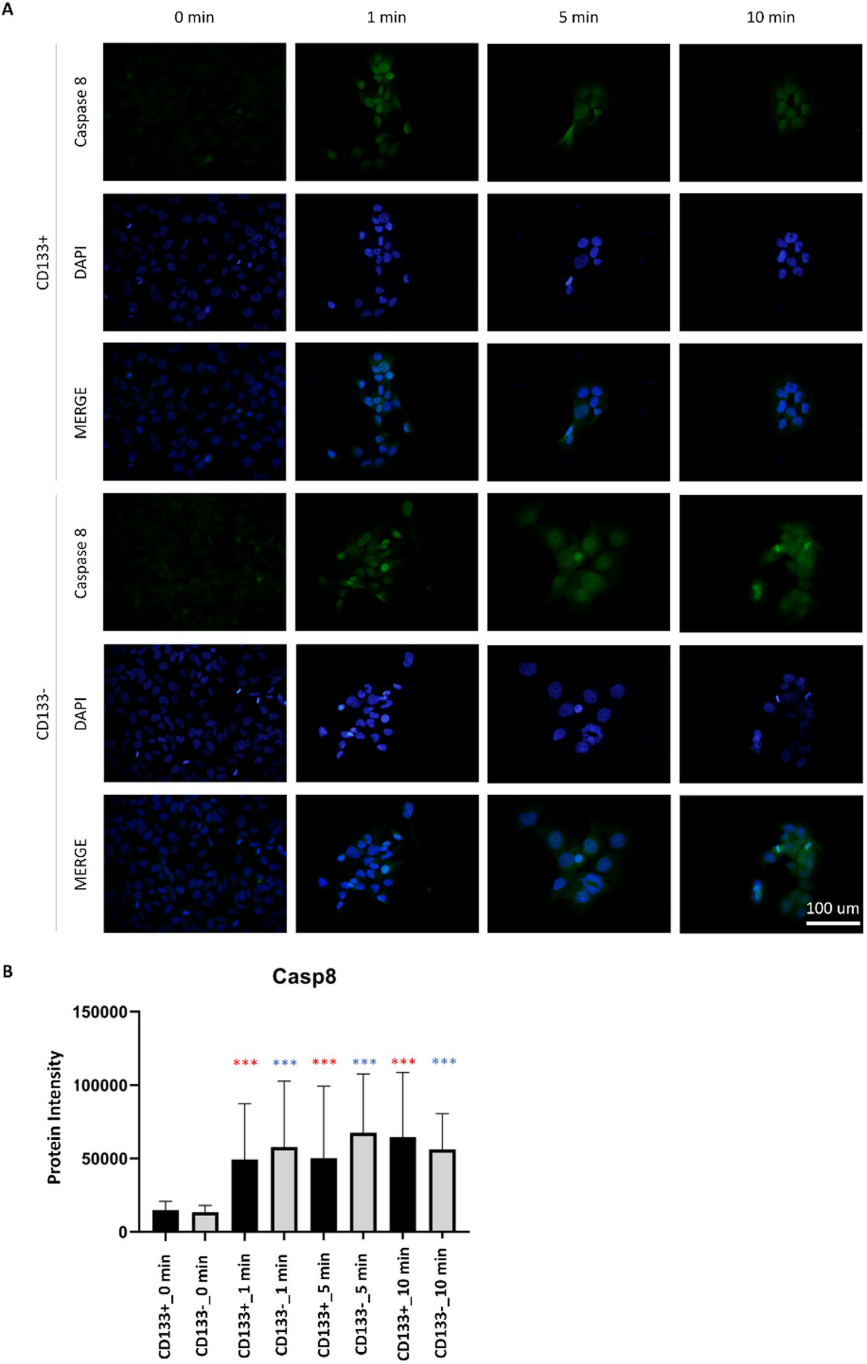
Casp8 protein immunostaining images (A) and quantitative analysis between groups (B). Group comparisons were made at each time point between CD133+ and CD133− populations (black asterisks), and within each group across timepoints compared to baseline (red for CD133+, blue for CD133−). All values represent mean ± SD. Statistical analysis was performed using one‐way ANOVA with Tukey's post hoc test. *p < 0.05, **p < 0.01, ***p < 0.001. No statistically significant difference was observed between the CD133+ and CD133− cell groups in the groups without LIPUS application. In the CD133+ cell groups, Casp8 levels increased compared to the control group (0 min) (*p* < 0.001), with the maximum increase observed at 5 min of application. A similar pattern was observed in the CD133− cell group, with an increase in Casp8 protein immunostaining within the cells following LIPUS application (*p* < 0.001), with the maximum increase recorded at 5 min of application.

**FIGURE 4:**
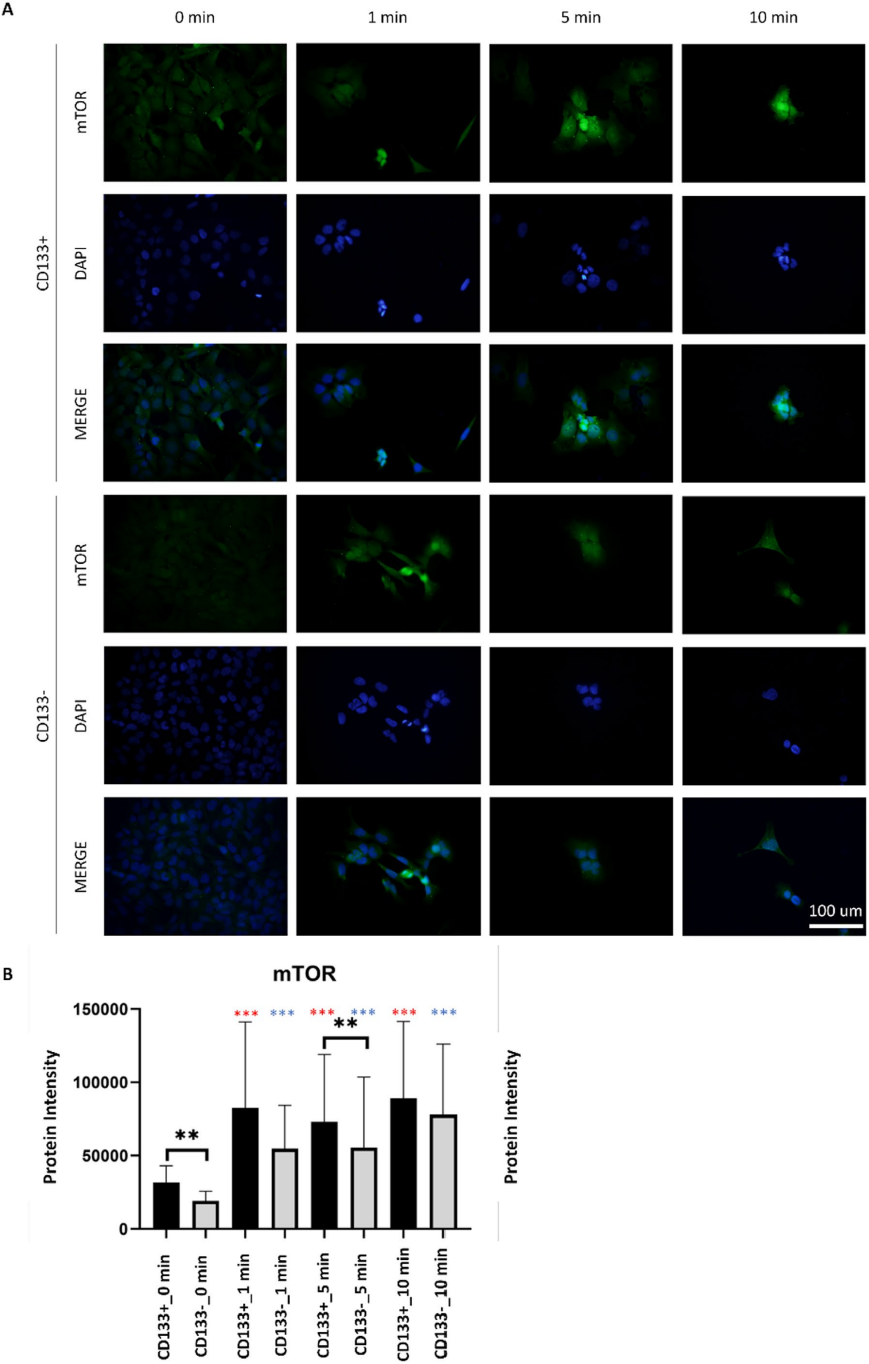
mTOR protein immunostaining images (A) and comparative quantification between groups (B). Comparisons were conducted between CD133+ and CD133− cells at each time point (black asterisks), and relative to the 0‐min control within each group (red asterisks for CD133+, blue asterisks for CD133−). Notably, a significant intergroup difference was observed only at the 5‐min application time. Data are expressed as mean ± SD. One‐way ANOVA and Tukey's post hoc test were used for statistical evaluation. *p < 0.05, **p < 0.01, ***p < 0.001. There was a statistically significant difference observed between the CD133+ and CD133− cell groups in the untreated control groups (*p* < 0.01). Among the LIPUS applications, a significant difference was only detected between the cell groups at 5 min of LIPUS application (*p* < 0.01). mTOR levels in CD133+ cell groups increased compared to the control group (0 min) (*p* < 0.001). A similar pattern was observed in the CD133− cell group. The immunostaining of mTOR protein inside the cells increased with LIPUS application (*p* < 0.001).

**FIGURE 5:**
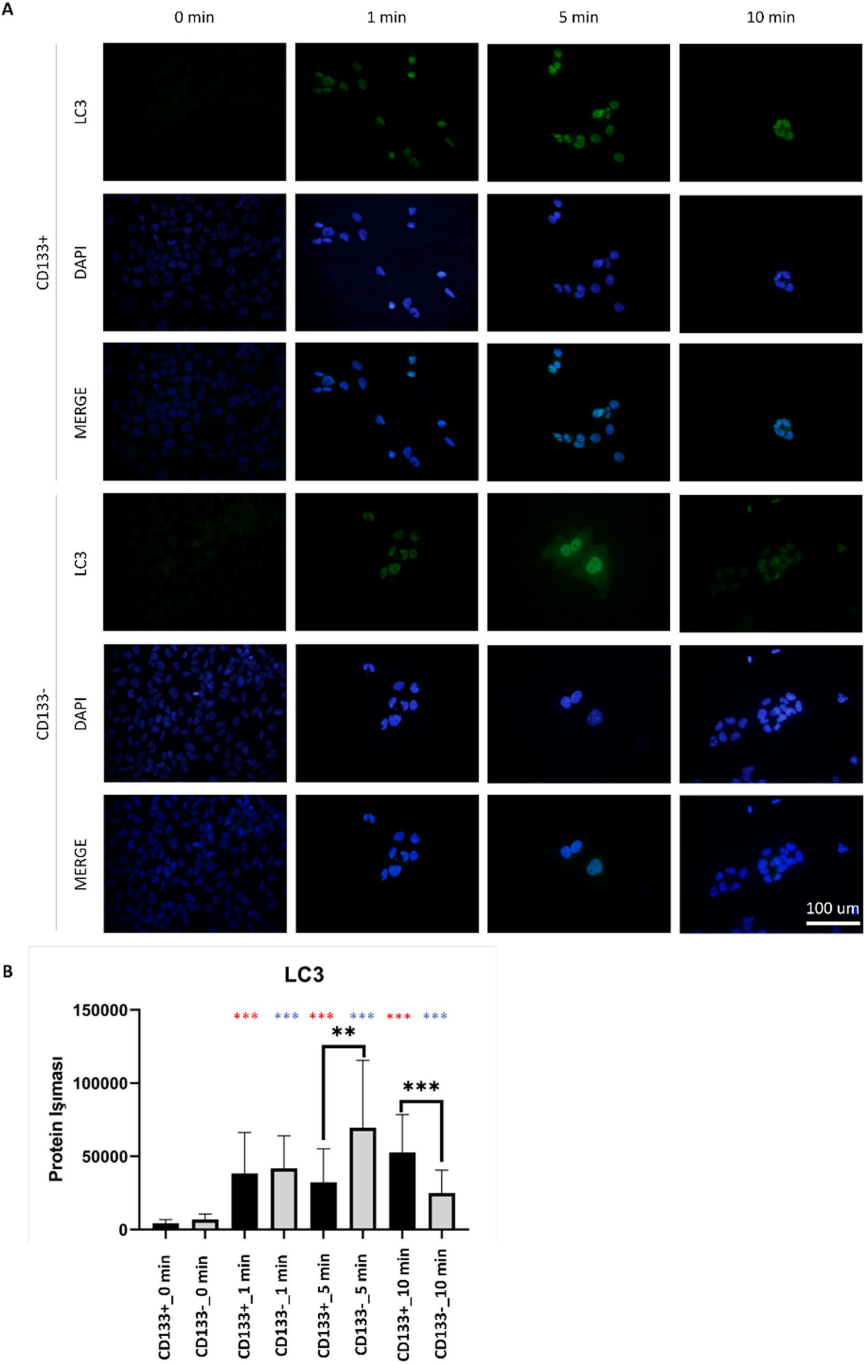
LC3 protein immunostaining images (A) and corresponding quantitative analysis (B). Comparisons were made within each group across LIPUS application times versus baseline (red asterisks for CD133+, blue for CD133−), and between CD133+ and CD133− groups at each time point (black asterisks). Significant differences were observed at 5 and 10 min. Data are represented as mean ± SD. Statistical analysis was conducted via one‐way ANOVA with Tukey's post hoc correction. *p < 0.05, **p < 0.01, ***p < 0.001. There was no statistically significant difference observed between the CD133+ and CD133− cell groups in the untreated control groups. However, a significant difference was detected between the cell groups at 5 min and 10 min of LIPUS application (*p* < 0.01 and *p* < 0.001, respectively). Following LIPUS application, the cells exhibited higher LC3 protein immunostaining compared to the control group (0 min). As a trend, LC3 immunostaining was higher in the CD133− cell group, but at 10 min of LIPUS application, it was even higher in the CD133+ cell group.

LIPUS application significantly increased mTOR protein intensity in both CD133+ and CD133− groups at 1, 5, and 10 min compared to baseline (*p* < 0.001) (Figure 3). In the CD133+ group, the highest increase was observed at 10 min, whereas the CD133− group followed a similar pattern. At baseline, mTOR levels were significantly higher in the CD133+ group compared to the CD133− group (*p* < 0.01). After LIPUS application, a significant difference between the groups was observed only at 5 min, with higher levels in the CD133+ group (*p* < 0.01). In the CD133+ group, LC3 fluorescence significantly increased at 1, 5, and 10 min after LIPUS application (*p* < 0.001), with the highest level at 10 min (Figure 4). Similarly, the CD133− group showed a significant increase (*p* < 0.001), peaking at 5 min. At baseline, there was no significant difference between the two groups (*p* > 0.05). However, after LIPUS application, significant differences were observed at 5 and 10 min. At 5 min, the CD133− group had higher LC3 levels, while at 10 min, the CD133+ group showed higher levels (*p* < 0.01 and *p* < 0.001, respectively) (Figure 5).

## 4 Discussion

Malignant melanoma, although less common than other types of skin cancer, is the most aggressive and high‐mortality skin cancer [31]. In recent years, as the global population has aged, the incidence of MM has also been on the rise worldwide. The etiopathogenesis of MM involves the inactivation of tumour suppressor genes or the activation of proto‐oncogenes. Additionally, mutations in proteins involved in intracellular signalling pathways such as NRAS/MAPK, PI3K/AKT (e.g., RAS, RAF, PTEN) can lead to the development of MM. As the depth of the tumour increases, the likelihood of metastasis also rises. Early detection and treatment of MM are crucial for patient survival. However, even in cases where early diagnosis is made and surgical excision is performed, there can still be recurrences in the early stages [32]. Furthermore, climate change is increasingly recognised as a significant environmental factor contributing to the rising incidence of melanoma, as prolonged sun exposure and UV radiation intensify, which may promote the activation of CSCs responsible for tumour initiation, resistance to treatment, and recurrence. The heterogeneous cellular composition of MM complicates the prediction of its biological behaviour. As a result, adjuvant chemotherapy has become increasingly important. However, treatment is often limited by drug resistance, high costs, and dose‐related side effects. These findings are consistent with the concept that biophysical cues, such as ultrasound‐induced vibrations, can alter apoptosis and autophagy signalling in cancer cells.

CSCs resemble normal stem cells in self‐renewal capacity but are distinct due to their resistance to radiation, chemotherapy, and apoptosis [6]. Certain markers are utilised to identify these cells. In studies, the CD133+ surface marker has been found to be abundantly present in stem cell‐like cells isolated from melanoma patients [10]. Melanoma, due to its relatively high abundance of CSCs compared to other cancer types, serves as a promising option for CSC research [11].

This interaction between cells and their physical environment lies at the core of mechanobiology. Mechanical cues such as tension, compression, and vibration can modulate stemness, apoptosis, and autophagy [12]. These mechanical forces include tension, loading, compression, vibration, pressure, and sound waves. Stem cells can respond to these forces by proliferating, migrating, self‐renewing, or differentiating depending on their current state [14]. Ultrasound is commonly used in the field of medicine for both imaging and therapeutic purposes. When used for therapeutic purposes, it is referred to as therapeutic ultrasound [33]. Electric energy causes the crystals in the ultrasound device's probe (head) to vibrate, thus generating sound waves. Frequencies typically used in therapy range from 0.7 to 3.3 MHz [15]. The energy intensity applied for the effect of therapeutic ultrasound is crucial, and as the intensity increases, the likelihood of causing damage to tissues also increases [34]. Due to its low intensity, LIPUS does not damage tissues, and therefore, it can be used for dental and bone healing and regeneration [35]. In recent years, the use of low‐intensity ultrasound for cancer treatment has gained importance. It induces sonoporation in the cell membrane, increasing the entry of chemotherapeutics into the cell or initiating apoptosis by causing the release of free radicals through cavitation effects, leading to the death of cancer cells [15, 36]. The activation of apoptosis and autophagy in cells is crucial in cancer treatment. In an in vivo study conducted by inducing melanoma in mice, therapeutic ultrasound applied to these mice showed an increase in apoptosis in melanoma cancer cells [37]. In another study involving the human hepatocellular carcinoma cell line, it was reported that LIPUS induced apoptosis in a dose‐dependent manner (at 0.5 W/cm^2^ and above) [23]. Furthermore, another study conducted using a mouse leukaemia cell line treated with ultrasound demonstrated the initiation of apoptosis and autophagy [38]. In general, studies in the literature involving LIPUS are related to the entry of cancer cells and used chemotherapeutic agents into the cells. There is limited research directly investigating the effects of LIPUS on CSCs. In cancer treatment, the eradication of CSCs is crucial to prevent tumour initiation, metastasis, and resistance to genomic‐targeted therapy, in addition to conventional chemotherapy. In an in vitro study involving ovarian cancer stem cells, it was found that LIPUS induced apoptosis in CSCs [39]. In contrast to this study, another study conducted in both in vivo and in vitro settings with human breast CSCs reported that ultrasound application alone had no significant effect compared to the control group [40]. However, in the study conducted by Guo et al., the applied ultrasound doses were in the range of 0.5–1.0–2.0–3.0–3.5–4.0 W/cm^2^, with application durations of 1–5–10 s. Unlike other studies, there is no standardised intensity level and application duration for LIPUS. Therefore, when interpreting the results, the intensity and application duration of LIPUS should be taken into consideration. In most studies, the application duration was kept constant while various doses were investigated [23, 41]. In our study, unlike the general approach, we kept the application dose constant and investigated the effects of three different application durations. Consistent with these findings, our results demonstrate that LIPUS induces both apoptotic and autophagic activity in MM cells and CSCs. Notably, this response was both time‐dependent and cell type‐specific.

Apoptosis is a programmed cell death process in the organism, aiming to maintain homeostasis. Losing control and failure of apoptosis can lead to the development of cancer. Apoptosis can be initiated through two main pathways, intrinsic and extrinsic. The intrinsic pathway is the mitochondrial pathway, where Casp3 activation occurs at the end of the cascade. The extrinsic pathway, on the other hand, leads to Casp8 activation [42]. In studies investigating the effects of LIPUS on various cancer cells, it has been reported that the Casp3 pathway is activated to initiate apoptosis [23, 43]. With mouse melanoma cells, it has been demonstrated that Casp3 activity increased after ultrasound application [44]. We did not come across many studies in the literature that assessed Casp8 after LIPUS application. However, on a pancreatic cancer cell line exposed to ultrasound, it was reported that Casp8 activity did not show statistically significant changes. In this study, they reported that when they created a microbubble effect with ultrasound, Casp8 significantly decreased, and this was related to necroptosis [43]. Here, we found that after LIPUS application, Casp3 and Casp8 activities increased in both cell lines compared to baseline. Unlike the literature, when comparing both cell lines, Casp3 activity was higher in the CD133− group after 1 min of LIPUS application and in the CD133+ group after 5 min of LIPUS application. However, no significant difference was observed between the two groups in terms of Casp8 activity. According to this result, it can be concluded that LIPUS activates apoptosis in both groups, but it may be more effective in MM cancer stem cells when applied for a longer duration.

Autophagy is triggered under conditions of cellular stress, such as nutrient scarcity, oxidative damage, and pathogen infection. Loss of control over autophagy can lead to cancer [45]. Autophagy can have a dual effect in cancer cells, acting as either a tumour suppressor or a tumour promoter, depending on the type, stage, and genetic characteristics of the cancer [46]. mTOR forms the mTORC1 complex, and inhibition of mTORC1 activates autophagy through the dephosphorylation of proteins that support the initiation of autophagy and the formation of autophagosomes [47]. In other words, mTOR is a negative regulator of autophagy [48]. LC3 is required for the elongation and maturation of the autophagosome through the protein complex it forms. Additionally, LC3 is commonly used to monitor autophagic activity [49]. Studies have shown that LIPUS application can affect autophagy [50]. In a study by Zeng et al., it was demonstrated that LIPUS could activate mTOR at low doses and inhibit it at high doses in lung cancer cells. The same study reported that LC3 was inhibited at low doses of LIPUS application and activated at high doses [51]. In our study, an increase in mTOR and LC3 fluorescence was observed in both MM cancer cell lines and MM cancer stem cell lines after LIPUS application compared to baseline. As the application duration increased, a fluctuation in mTOR activity was observed in MM cancer stem cell lines, while LC3 activity steadily increased, with a greater increase observed in the MM cancer cell line after 10 min of application. mTOR and LC3 may vary temporally and in terms of autophagy diversity. In this case, it is considered that mTOR and LC3 may play roles in compensatory mechanisms for autophagy.

Overall, this study provides novel insights into the time‐sensitive modulation of cell death pathways by LIPUS in melanoma CSCs. These findings support the potential of LIPUS as a non‐invasive therapeutic strategy targeting treatment‐resistant cancer stem cells.

## 5 Conclusions

In summary, LIPUS application induced time‐dependent activation of apoptosis and autophagy markers in both CD133+ melanoma stem cells and CD133− non‐stem cells. The delayed but sustained response observed in CSCs suggests a differential sensitivity to mechanical stimulation compared to non‐stem cells. This study makes a significant contribution to cancer mechanobiology by demonstrating that ultrasound‐induced mechanical stimulation can modulate cell death pathways in therapy‐resistant populations such as cancer stem cells. Furthermore, it enhances our understanding of the biophysical effects of low‐intensity ultrasound on CSCs, paving the way for the development of mechanistically guided, non‐invasive therapeutic approaches.

## Author Contributions

**Omer Dikici:** conceptualization (equal), data curation (equal), formal analysis (equal), funding acquisition (equal), investigation (equal), methodology (equal), project administration (equal), resources (equal), software (equal), supervision (equal), validation (equal), visualization (equal), writing – original draft (equal), writing – review and editing (equal). **Berrin Ozdil:** conceptualization (equal), data curation (equal), formal analysis (equal), funding acquisition (equal), investigation (equal), methodology (equal), project administration (equal), resources (equal), software (equal), supervision (equal), validation (equal), visualization (equal), writing – original draft (equal), writing – review and editing (equal). **Taha Kadir Yesin:** conceptualization (equal), data curation (equal), formal analysis (equal), funding acquisition (equal), investigation (equal), methodology (equal), project administration (equal), resources (equal), software (equal), validation (equal), visualization (equal), writing – original draft (equal), writing – review and editing (equal). **Aylin Dikici:** conceptualization (equal), data curation (equal), formal analysis (equal), funding acquisition (equal), investigation (equal), methodology (equal), resources (equal), software (equal), validation (equal), visualization (equal), writing – original draft (equal), writing – review and editing (equal). **Yasemin Adalı:** conceptualization (equal), data curation (equal), formal analysis (equal), funding acquisition (equal), investigation (equal), methodology (equal), project administration (equal), software (equal), visualization (equal), writing – original draft (equal), writing – review and editing (equal). **Huseyin Aktug:** conceptualization (equal), data curation (equal), formal analysis (equal), funding acquisition (equal), investigation (equal), methodology (equal), project administration (equal), resources (equal), software (equal), supervision (equal), validation (equal), visualization (equal), writing – original draft (equal), writing – review and editing (equal).

## Conflicts of Interest

The authors declare no conflicts of interest.

## Data Availability

The data that support the findings of this study are available from the corresponding author upon reasonable request.
